# Changes in Oxygen Metabolism Biomarkers of Ischemic Tissue Treated With Electrical Stimulation

**DOI:** 10.1161/SVIN.125.002094

**Published:** 2025-12-12

**Authors:** Mersedeh Bahr-Hosseini, Mona Asghariahmadabad, Marom Bikson, Jeffrey L. Saver, David S. Liebeskind, Kambiz Nael

**Affiliations:** Department of Neurology, David Geffen School of Medicine at UCLA, University of California, Los Angeles (M.B.-H., J.L.S., D.S.L.).; Department of Radiology and Biomedical Imaging, University of California, San Francisco (M.A., K.N.).; Department of Biomedical Engineering, The City College of New York (CCNY), NY (M.B.).

**Keywords:** cytoprotection, infarction, oxygen, perfusion, vasodilation

In the first-in-human proof-of-concept TESSERACT study (Transcranial Electrical Stimulation in Stroke Early After Onset Clinical Trial), delivering high-definition cathodal transcranial direct current stimulation (HD C-tDCS) to penumbral tissue was shown to be a feasible and tolerable treatment strategy for acute ischemic stroke.^1^ Additionally, in this dose-escalation study, signals of efficacy were observed: patients receiving active HD C-tDCS had a higher proportion of penumbral salvage (median, 66%) compared with sham patients (median, 0%) on standard hemodynamic perfusion imaging. The mechanisms through which HD C-tDCS may salvage penumbra are collateral enhancement via electrical current-induced pial arterial vasodilation and direct cytoprotection via inhibition of peri-infarct excitotoxicity.^2^

Additional imaging biomarkers that provide insight into ischemic tissue fate and therapeutic response are oxygen extraction fraction (OEF) and cerebral metabolic rate of oxygen (CMRO2).^3^ This report, for the first time, describes the changes in oxygen metabolism biomarkers of the penumbral tissue in patients enrolled in TESSERACT. We aimed to corroborate the effectiveness signals observed in the study and further investigate the HD C-tDCS mechanism of action as an acute ischemic stroke treatment strategy.

## Methods

Patients who had dynamic susceptibility contrast (DSC) MR perfusion imaging before, early (2 hours), and late (24 hours) poststimulation were included. The images were processed and analyzed to calculate OEF and CMRO2 within penumbral tissue and the contralateral nonischemic hemisphere.

Cercare Medical Neurosuite 15 was used to generate OEF and CMRO2 maps from DSC perfusion using a biophysical model for the vasculature.^4^ From the DSC signal, per-voxel cerebral blood flow (CBF) and transit-time distributions are derived, the latter from Mean Transit Time and capillary transit-time heterogeneity using a flexible gamma variate residue function adapted iteratively to each voxel through a Bayesian approach.^4^ The model assumes a fixed tissue oxygen tension (25 mm Hg) and estimates the maximal OEF (OEF^max^) by integrating the oxygen extraction efficiency across the per-voxel adaptively estimated transit-time distribution. After computing OEF^max^ voxel-wise, the CMRO₂ is computed as the product of OEF^max^, CBF, and arterial oxygen content.

Each individual’s penumbral tissue values were divided by contralateral nonischemic hemisphere values to acquire the normalized relative ratios.

## Results

Out of 10 study patients, 5 (4 active and 1 sham) who had DCS MR perfusion imaging at all 3 time points of prestimulation, early (2 hours), and late (24 hours) poststimulation were included in the analysis. Prestimulation, the penumbral OEF was elevated (median, 1.8 [interquartile range (IQR), 1.5–1.95]), and CMRO2 was slightly reduced (median, 0.8 [IQR, 0.7–1]) compared with the contralateral hemisphere in all 5 patients.

Following active stimulation, there was a reduction of OEF (median, 1.3 [IQR, 1–1.4]) and concomitant restoration and even enhancement of CMRO2 (median, 1.5 [IQR, 1.1–2]) to exceed that of the contralateral hemisphere after stimulation, indicating metabolic and cellular salvage^3^ (Figure).

**Figure.**
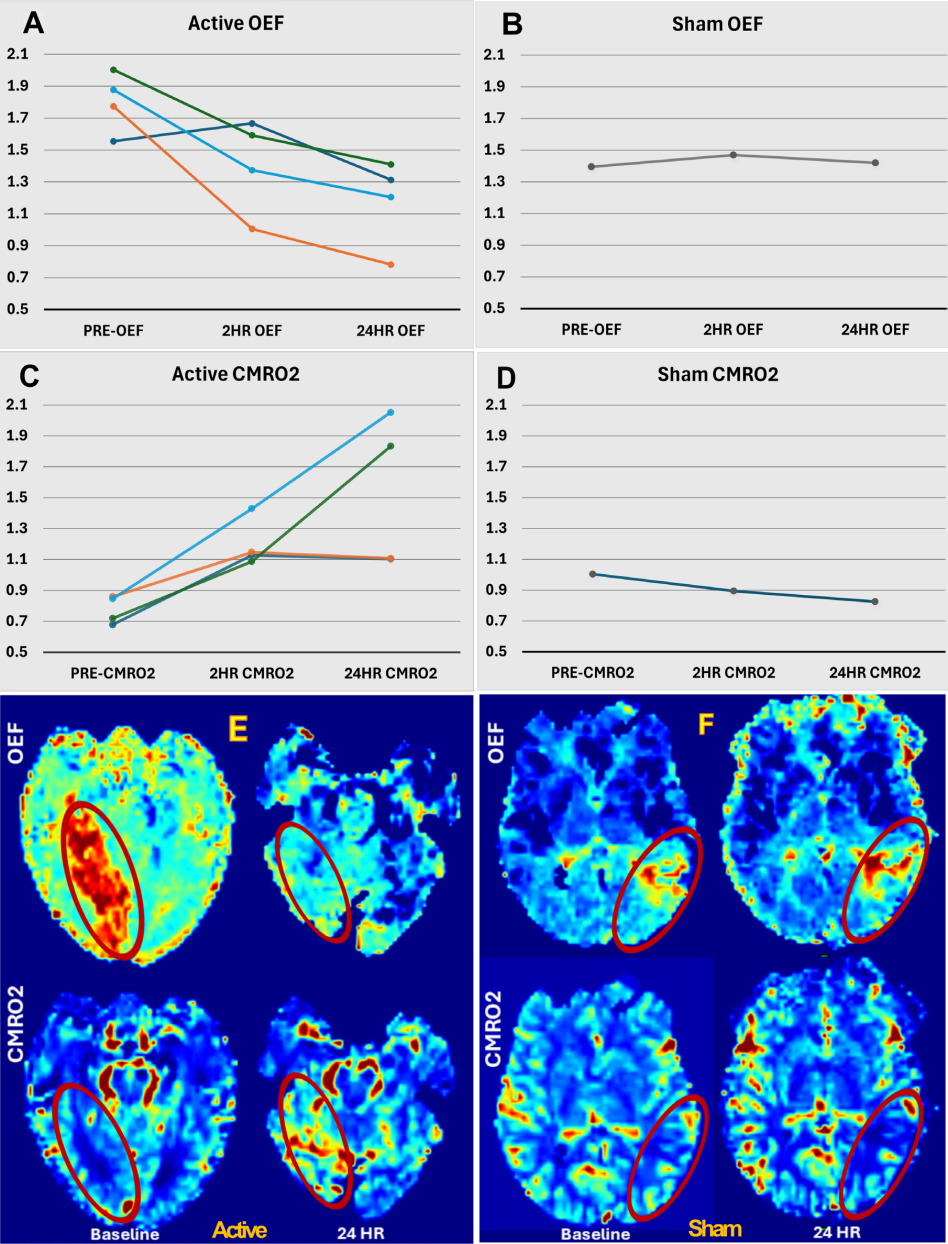
**Individual patient-level changes in oxygen metabolism biomarkers in active stimulation patients versus sham and depiction of color-coded oxygen metabolism maps in one exemplary active patient versus sham pre- to poststimulation. A**–**D**, Interval changes in oxygen extraction fraction (OEF) and cerebral metabolic rate of oxygen (CMRO2) in 4 active stimulation patients (**A** and **C**) compared with 1 sham (**B** and **D**) across 3 time points of baseline, 2-hour, and 24-hour poststimulation. The individual patient-level data points of OEF and CMRO2 are shown in ratios (vertical axes): mean values of ischemic tissue divided by the mean values of the nonischemic contralateral hemisphere. **E** and **F**, Changes in OEF (top row) and CMRO2 (bottom row) of ischemic tissue (circled in red) from baseline (left) to 24 hours (right) in 1 exemplary active patient (**E**) and sham (**F**). A more pronounced reduction in OEF and enhancement of CMRO2 from baseline to poststimulation are suggested in the active patient.

The median changes in OEF and CMRO2, from baseline to 2-hour poststimulation were −0.4 (IQR, −0.7 to −0.2) and +0.5 (0.4–0.6), respectively. From 2- to 24-hour poststimulation, median changes in OEF and CMRO2 of −0.2 (−0.3 to −0.2) and + 0.3 (0–0.7) were observed, respectively. The overall OEF and CMRO2 changes from baseline to 24-hour poststimulation were -0.6 (−0.9 to −0.4) and +0.8 (0.3–1.2), respectively.

On the other hand, in 1 sham individual, no significant change in overall OEF was observed, and CMRO2 further decreased and remained below that of the contralateral hemisphere, suggestive of infarction (Figure [F]).^3^

## Discussion

The pattern of metabolic changes in the ischemic tissue following active HD C-tDCS stimulation, characterized by a simultaneous normalization of oxygen extraction and restoration of oxygen metabolism poststimulation, suggests metabolic and cellular salvage. Furthermore, the enhancement of ischemic tissue CMRO2 beyond that of the nonischemic contralateral hemisphere is likely due to the primary vasodilatory effect of the electrical current, which enhances CBF and volume.^2^ As CMRO2 values are derived from CBF, mathematical coupling is the most likely explanation for the enhanced CMRO2 observed in our study. Muccio et al recently described CMRO2 and CBF enhancement in healthy volunteers undergoing simultaneous tDCS and magnetic resonance imaging, which was deemed related to primary neuronal activation in a coupled neurovascular system.^5^ Although our finding could similarly be attributed to the restoration of metabolism-flow coupling in the salvaged brain tissue, the reduction in OEF is more supportive of primary CBF enhancement as the driving force.

This report has several limitations. The sample size is small and the report has a descriptive nature, hence, our preliminary findings and their functional relevance require further validation in larger studies of HD C-tDCS on patients with acute ischemic as planned in phase 2 of the TESSERACT study. Furthermore, the metabolic variables reported are derived from DSC magnetic resonance imaging as opposed to the gold standard positron emission tomography imaging. However, given the infeasibility of obtaining positron emission tomography in hyperacute settings, DSC perfusion MR-derived oxygen metabolism biomarkers could serve as a viable strategy to obtain surrogate efficacy imaging biomarkers.

## ARTICLE INFORMATION

### Sources of Funding

The TESSERACT study (Transcranial Electrical Stimulation in Stroke Early After Onset Clinical Trial) was funded by an American Heart Association award (18CDA34110160).

### Disclosures

The Regents of the University of California hold a patent (No. 63/370,428) on Transcranial Electrical Stimulation in Stroke Early After Onset with Dr Bahr-Hosseini as inventor. Dr Bahr-Hosseini receives funding from the National Institute of Neurological Disorders and Stroke (NINDS; NIHUG3NS134619) for TESSERACT-2 (Transcranial Electrical Stimulation in Stroke Early After Onset Clinical Trial 2) as a principal investigator. The Regents of the University of California hold a patent (No. 63/370,428) on Transcranial Electrical Stimulation in Stroke Early after Onset with Dr Bikson as coinventor. The City University of New York holds patents on brain stimulation with Dr Bikson as inventor. Dr Bikson has equity in Soterix Medical Inc. Dr Bikson receives funding from NINDS (NIHUG3NS134619) for TESSERACT-2 as a coinvestigator. The Regents of the University of California hold a patent (No. 63/370,428) on Transcranial Electrical Stimulation in Stroke Early after Onset with MB as coinventor. Dr Saver receives funding from NINDS (NIHUG3NS134619) for TESSERACT 2 as a coinvestigator. Dr Liebeskind receives funding from NINDS (NIHUG3NS134619) for TESSERACT-2 as a coinvestigator.

Dr Nael receives funding from NINDS (NIHUG3NS134619) for TESSERACT-2 as a coinvestigator. The other author reports no conflicts.
